# From Genetics to Functional Genomics: Improvement in Drought Signaling and Tolerance in Wheat

**DOI:** 10.3389/fpls.2015.01012

**Published:** 2015-11-19

**Authors:** Hikmet Budak, Babar Hussain, Zaeema Khan, Neslihan Z. Ozturk, Naimat Ullah

**Affiliations:** ^1^Plant Genomics Group, Molecular Biology, Genetics and Bioengineering Program, Faculty of Engineering and Natural Sciences, Sabanci UniversityIstanbul, Turkey; ^2^Department of Agricultural Genetic Engineering, Faculty of Agricultural Sciences and Technologies, Niǧde UniversityNiǧde, Turkey

**Keywords:** wheat, ABA, drought, signaling, functional genomics, transcription factors, transcriptomics

## Abstract

Drought being a yield limiting factor has become a major threat to international food security. It is a complex trait and drought tolerance response is carried out by various genes, transcription factors (TFs), microRNAs (miRNAs), hormones, proteins, co-factors, ions, and metabolites. This complexity has limited the development of wheat cultivars for drought tolerance by classical breeding. However, attempts have been made to fill the lost genetic diversity by crossing wheat with wild wheat relatives. In recent years, several molecular markers including single nucleotide polymorphisms (SNPs) and quantitative trait loci (QTLs) associated with genes for drought signaling pathways have been reported. Screening of large wheat collections by marker assisted selection (MAS) and transformation of wheat with different genes/TFs has improved drought signaling pathways and tolerance. Several miRNAs also provide drought tolerance to wheat by regulating various TFs/genes. Emergence of OMICS techniques including transcriptomics, proteomics, metabolomics, and ionomics has helped to identify and characterize the genes, proteins, metabolites, and ions involved in drought signaling pathways. Together, all these efforts helped in understanding the complex drought tolerance mechanism. Here, we have reviewed the advances in wide hybridization, MAS, QTL mapping, miRNAs, transgenic technique, genome editing system, and above mentioned functional genomics tools for identification and utility of signaling molecules for improvement in wheat drought tolerance.

## Introduction

Global warming has resulted in decreased precipitation and increased evaporation, causing more frequent drought spells worldwide. Drought reduces the plant yield up to 50% which is a great economic loss for the farming community (Akpinar et al., 2013). Consequently, development of drought tolerant wheat cultivars has become a serious challenge for the plant breeders to ensure the food security of the masses (Budak et al., 2013a). Drought is a multifaceted trait; plant responses to drought are affected by various factors including growth conditions, physiology, genotype, developmental stage, drought severity, and duration. Thus, drought tolerance mechanisms involve diverse gene expression patterns and as complex signaling pathways (Kantar et al., 2011a; Akpinar et al., 2012). Bread wheat is an important staple food worldwide, therefore efforts have been made to develop drought tolerant varieties (Budak et al., 2013b). Drought signaling pathways involve crosstalk among various biomolecules which makes breeding for drought tolerance an uphill task (Akpinar et al., 2012). In recent years, genomics knowledge based on Next Generation Sequencing (NGS), gene editing systems (Shan et al., 2013), gene silencing (Yin et al., 2014), and over-expression methods (Saad et al., 2013) have increased our understanding about drought signaling pathways. At the transcriptome level, the RNA deep sequencing (Akpinar et al., 2015) and microarray analyses (Ergen et al., 2009) are employed to elucidate the differential expression of RNA transcripts involved in drought response. Moreover, microRNAs (miRNAs; Budak and Akpinar, 2015), hormones (Reddy et al., 2014), quantitative trait loci (QTLs; Barakat et al., 2015), metabolites (Xiao et al., 2012), transcription factors (TFs), and drought-related proteins (Lucas et al., 2011a; Alvarez et al., 2014) are key players in drought signaling. These factors regulate the gene expression in response to drought. TFs also interact with plant stress hormones, e.g., abscisic acid (ABA), jasmonic acid (JA), and salicylic acid (SA) in mediating drought response (Nakashima et al., 2014). To elucidate complex wheat drought signaling which will help in developing improved varieties, powerful tools are required for multiplexed or simultaneous detection of signaling molecules. Advances in functional genomics tools have provided us the opportunity to detect above mentioned molecules with ease, efficacy and accuracy thus opening a new era of crop improvement (Colmsee et al., 2012). In this review, we have summarized the advances in genetics, genomics, and functional genomics for identification of novel genes and their subsequent use in breeding programs for improved drought tolerance.

## Signaling Pathways in Wheat for Drought Tolerance

Drought signaling is categorized into ABA-dependent and ABA-independent pathways as ABA is the first line of defense against drought. ABA-dependent signaling consists of two main gene clusters (regulons) regulated by ABA-responsive element-binding protein/ABA-binding factor (the AREB/ABF regulon) and the MYC/MYB regulon. Previous studies have shown that AP2/EREBP (ERF) TFs are engaged in both ABA-dependent and independent signaling pathways. Despite being two distinct and independent pathways, there is plausibly some crosstalk between both (Lata and Prasad, 2011). The AP2/ERF TFs family includes the ethylene-response factors, e.g., a TaERF promotes drought tolerance in wheat with increased proline and chlorophyll levels (Rong et al., 2014). The sucrose non-fermenting1-related protein kinase 2 family (SnRK2) consists of plant specific Ser/Thr kinases which are positive regulators of ABA signaling. SnRK2s were first reported to be involved in ABA signaling in wheat (PKABA1; Fujii and Zhu, 2012). The SnRK TFs are also involved in ABA independent pathway (Lata and Prasad, 2011). Although not specifically studied in wheat, the ABA-dependent pathways in rice and *Arabidopsis* have been extensively analyzed (Todaka et al., 2012). The ABA-independent regulons include the CBF/DREB (cold-binding factor/dehydration responsive element binding), NAC, and ZF-HD (zinc-finger homeodomain; Lata and Prasad, 2011). The transcriptional regulatory network based on DREBs is induced by dehydration in wheat. There are two known DREB regulons; DREB1/CBF and DREB2 (Edae et al., 2013). Above mentioned signaling pathways and their roles in drought tolerance have been extensively discussed in this review.

## Genetics Based Improvement in Drought Signaling

The selection of an appropriate breeding strategy to develop drought tolerant cultivars is the key step for a successful breeding program. Therefore, strategies which can transfer specific genes, exploit wild relatives of crops, identify and transfer genes with ease, require less time, and labor to develop cultivars are of great value (Hussain, 2015). The advances in genetics, genomics, and functional genomics have enabled researchers to combine one or more advantages of different strategies to develop drought tolerant wheat. Here, we have described the advances in these methods.

### Classical Breeding for Improving Drought Tolerance in Wheat

Classical breeding dates back to 5,000–10,000 years when man domesticated selective plant species based on their better taste. Domestication was followed by selection of high yielding genotypes and cross-hybridization to recombine tolerance genes from different sources (Hussain, 2015). The presence of genetic variation in wheat is the key to identify the contrasting parents for classical breeding (Shelden and Roessner, 2013). Significant genetic variation in wheat for drought tolerance has been identified for selection of diverse parents. Cross-hybridization of wheat diploid progenitors produce drought tolerant synthetic hexaploid (SHs) wheat (see the section Introgression of Drought Tolerance Genes from Wild Species) with introgression of several novel drought tolerance genes (Zhang et al., 2005). Most of plant breeders selected the drought and other stress tolerant wheat varieties on the basis of higher yields and ignored the physiological mechanism behind it. Therefore, few cultivars having drought and abiotic stress tolerance have been developed in comparison to the ones improved for high yield (Hussain et al., 2015). Under drought, plant machinery shifts its focus to ABA production for downstream activation of signaling and tolerance mechanisms which lowers the grain filling and yield. Therefore, the balance between yield and drought tolerance needs to be investigated (Alvarez et al., 2014). ABA content has been used as selection index for screening wheat under drought and contrasting parents for its production have been crossed. Several QTLs and genes involved in signaling pathways have been identified in subsequent segregating populations (Iehisa et al., 2014; see the section QTL Mapping for Drought Signaling Genes in Wheat). Longer time, intensive labor requirements, transfer of non-desirable genes, limited resistance resources, genetic barriers and limited understanding of tolerance mechanisms are problems associated with classical breeding (Hussain, 2015). Since breeding to date has not been focused on signaling pathways, there is a need to combine the available information collected by QTLs, MAS, and Omics tools with traditional traits for improved wheat drought tolerance.

### Introgression of Drought Tolerance Genes from Wild Species

Significant loss of genetic diversity has occurred at three levels: (a) Species level (domestication), (b) Varietal level (green revolution), and (c) Gene level (breeding cycles). High yielding wheat developed through green revolution has less stress tolerance (Hussain, 2015). It is the time for plant breeders to look back and utilize this lost genetic diversity as some wild wheat relatives are potential sources of drought tolerance. For example, wild emmer wheat (*Triticum dicoccoides*) has inter and intra-varietal genetic diversity for water use efficiency (WUE), phenology, and contains several genes and QTLs for drought tolerance (Nevo and Chen, 2010). Gene expression studies in emmer wheat identified over 13,000 expressed sequence tags (ESTs) in response to drought (Ergen and Budak, 2009), and 33 outlier loci for drought tolerance were identified by single nucleotide polymorphisms (SNPs) markers (Ren et al., 2013). Transcriptomic analysis identified several genes and TFs involved in ethylene, IP3, and ABA dependent signaling pathways in wild emmer wheat (Ergen et al., 2009). SHs wheat constituted by crossing these wild relatives gained many novel QTLs and genes for ABA responsiveness and signaling (Iehisa et al., 2014). Role of *DREBs* in conferring drought tolerance to *T. dicoccoides* has also been established (Lucas et al., 2011b).

Introgression of drought tolerance to cultivated wheat from *Aegilops tauschii* was achieved by crossing it with durum wheat to make SHs wheat. DNA fingerprinting of SHs showed high genetic diversity with longer roots and higher soluble carbohydrates to resist water deficiency (Reynolds et al., 2007). D genome of *A. tauschii* contains several drought responsive genes potentiating the development of drought tolerant SHs wheat through crossing. *A. tauschii* and related SHs showed significant variation for ABA responsiveness when gene expression analyses were performed for ABA inducing *WABI5* and three downstream Cor/LEA protein coding genes (*Wrab18, Wrab17*, and *Wdhn13*) while the line with enhanced expression of *Wdhn13* showed salt and dehydration tolerance (Iehisa and Takumi, 2012). Proteomics approach has identified several proteins involved in ABA signaling (ABA 8′-hydroxylase, MPK6, dehydrin, 30S ribosomal protein S1, retrotransposon protein, a 70 kDa HSP) in the wild wheat relative, *Kengyilia thoroldiana* under drought. Proteins involved in antioxidative enzyme activity (thioredoxin peroxidase, ascorbate peroxidase, Cu/Zn superoxide dismutase) also showed increased expression levels (Yang et al., 2015). The value of wild wheat relatives as donors of drought genes has not only been established on morphological bases, but also validated with genomics (QTLs) and functional genomics (transcriptomics, proteomics, ESTs, SNPs) tools. Furthermore, their utility as drought gene donors has been confirmed in SHs. Therefore, we suggest that plant breeders should focus on wheat wild relatives to enhance the genetic diversity of wheat for drought tolerance.

### Molecular Markers for Identification of Drought Signaling Genes

Selection in classical breeding is performed on the basis of morphological and economical traits which are highly influenced by the environment. Environmental influence on phenotypic expression creates confusion in selection of desirable traits. Discovery of DNA markers for economic and stress related crop traits have helped to select the desirable traits and parents with ease, efficacy and reliability in remarkably shorter time. Therefore markers, especially the SNPs have added more power to identify the genes linked to drought and other stresses (Budak et al., 2013b; Hussain, 2015). DNA markers for various genes involved in drought signaling have been reported, e.g., RAPD markers by using P21F/P21R and P25F/PR primers in A genome; and P18F/P18R primer in B genome mapped *DREB1* on 3A chromosome (Huseynova and Rustamova, 2010). In wheat, DREBs were tagged with five SNPs in A (P21F/P21R and P25F/PR primers), B (P18F/P18R primer), and D genome (P20F/P20R and P22F/PR primers). *DREB1* gene was tagged on chromosome 3A, 3B, and 3D. S646 and S770 SNPs were used and SNP S770 mapped *DREB-B1* between markers *Xfbb117* and *Xmwg818* on chromosome 3BL (Wei et al., 2009). SNPs identified the involvement of five signaling genes in yield and drought tolerance pathways. The *DREB1A* correlated with heading date, vegetation index and biomass, while flag leaf width, harvest index and leaf senescence were associated with *ERA1-B* and *ERA1-D* (enhanced response to ABA) genes. Other signaling genes, *1-FEH-A* and *1-FEH-B* (fructan-1-exohydrolase) were linked to yield and thousand kernel weight (Edae et al., 2013). Significant relationships between morpho-physiological traits and SNPs suggested key role of detected SNPs in drought tolerance.

High resolution melting (HRM) technology is the most powerful tool to identify the allelic variations. Use of HRM found that allelic variation in *DREB* TFs identified by SNPs led to variation in peptide sequences as well. The variation in peptide sequences was linked with differences in protein geometry and recognition of *cis*-elements involved in ABA signaling (Mondini et al., 2015). Two important TFs, i.e., *DREB1, WRKY1*, and a Na^+^ transporter, *HKT-1* conferring drought and salt tolerance were also mapped by SNPs (Mondini et al., 2012). SNPs were used to map *TaSnRK2.8* gene which plays important role in carbohydrate metabolism, protein–protein interaction, and ABA signaling (Zhang et al., 2013). Chromosome locations and primers for these markers are given in **Table 1**. It can be concluded that MAS has a lot of promise to identify the signaling genes. Strong association between signaling genes and drought related physiological traits suggest that MAS should be focused to identify the signaling genes (Edae et al., 2013). This can help to improve the drought tolerance with less effort, time and resources and can speed up the breeding programs in future.

**Table 1:** Molecular markers identified for wheat drought signaling genes.

Marker type	Primer	Chromosome location	Target drought signaling gene	Reference
SNP	P21F/P21R and P25F/PR	3A	*DREB1*	Wei et al., 2009
SNP S770	P18F/P18R	3BL *Xfbb117-Xmwg818*	*DREB1*	Wei et al., 2009
SNP	P20F/P20R and P22F/PR	3D	*DREB1*	Wei et al., 2009
RAPD	P25F/PR	3A	*DREB1*	Huseynova and Rustamova, 2010
RAPD	P18F/P18R	3A	*DREB1*	Huseynova and Rustamova, 2010
SNP	DREB1a and DREB1b	U16709.1^∗^	*DREB1*	Mondini et al., 2012
SNP	WRKY1	DQ323885.1^∗^	*WRKY1*	Mondini et al., 2012
SNP	HKT-1	AF303376.1^∗^	*HKT-1*	Mondini et al., 2012
SNP	P21	3A	*DREB1A*	Edae et al., 2013
SNP	ERA1B	3A, 3B, 3D	*ERA1-B*	Edae et al., 2013
SNP	ERA1D	–	*ERA1-D*	Edae et al., 2013
SNP	W12	6A	*1-FEH-A*	Edae et al., 2013
SNP	W32	–	*1-FEH-B*	Edae et al., 2013
SNP	M13	5A	*TaSnRK2.8*	Zhang et al., 2013
SNP	DREB2a and DREB2b	–	*DREB2*	Mondini et al., 2015
SNP	DREB3a and DREB3b	–	*DREB3*	Mondini et al., 2015

*^∗^Ref gene.*

### Transgenic Approaches for Improving Drought Signaling

Loss of tolerance genes by genetic erosion should be filled with such efficient and reliable methods that can transfer genes in a short time. Recombinant DNA technology has emerged as a powerful tool for the purpose. It provides the additional benefit of having no genetic barriers, thereby; so can transferring the genes from any wild relative, land race or other species (Hussain, 2015). Candidate genes for drought tolerance in wheat include TFs which regulate the signaling genes, genes encoding defense molecules [Reactive oxygen species (ROS), proline, JA, SA], and for production of defense proteins (Yang et al., 2010). Here, we have described the major achievements in wheat (see summary in **Table 2**).

**Table 2:** Improvement in wheat drought signaling and tolerance by transgenic approaches.

Transformed gene	Improvement in signaling and tolerance	Reference
*HVA1*	Abscisic acid (ABA) signaling, produce Late embryogenesis abundant 3 (LEA3) for cell membrane integrity, higher biomass production and water use efficiency (WUE), drought, and salt tolerance	Sivamani et al., 2000
*HVA1*	ABA responsiveness, ABA signaling, higher WUE and relative water content (RWC), stable yield, drought tolerance	Bahieldin et al., 2005
*GmDREB*	ABA independent signaling (AIS), drought, and salt tolerance	Shiqing et al., 2005
*GmDREB*	AIS, 2-fold higher proline production, stay green, SURV, drought tolerance	Wang et al., 2006
*GhDREB*	AIS, higher soluble sugars and chlorophyll production, improved drought, salt, and freezing tolerance	Gao et al., 2009
*DREB1A*	Higher SURV, WUE, and yield under drought	Pierre et al., 2012
*SNAC1*	Activation of sucrose phosphate synthase, type 2C protein phosphatases and 1-phosphatidylinositol-3-phosphate-5-kinase genes for ABA signaling, high RWC, chlorophyll content and biomass, enhanced salinity and drought tolerance	Saad et al., 2013
Alfalfa aldose reductase	Antioxidant defense, ABA signaling, detoxification of aldehyde substrate, green biomass production, drought tolerance	Fehér-Juhász et al., 2014

#### Drought Signaling by Introducing *DREBs*

A soybean based DREB gene (*GmDREB*; Accession No. AF514908) was transformed to wheat by gene gun bombardment using ubiquitin and RD29A promoters, and transgenic plants with both promoters showed increased drought and salt tolerance (Shiqing et al., 2005). This increased tolerance of the crop was linked with to twofold higher proline production, stay green phenomenon under drought and survival and recovery on re-watering (SURV) after drought spell (Wang et al., 2006) suggesting a role of signaling pathway in downstream proline production. Transformation of wheat with a cotton originating DREB (*GhDREB*) improved drought, salt, and freezing tolerance due to higher production of soluble sugars and chlorophyll in leaves (Gao et al., 2009). Transgenic wheat with *DREB1A* was subjected to field screening on the basis of SURV and WUE. Although the event was selected in greenhouse, plants showed even higher yield under field drought (Pierre et al., 2012). However, there is dire need to find out activated genes or expressed proteins by these TFs to fully understand their role in signaling pathways.

#### Drought Signaling by Introducing *HVA1* Gene

Various studies aimed to find the function of ABA in regulating the expression of drought tolerance genes. A number of such genes code for the proteins involved in stomata closure to check the transpiration against the cell dehydration. Late embryogenesis abundant 3 (LEA3) in barley is one of such protein encoded by *Hordeum vulgare* abundant protein 1 (*HVA1*) gene. This gene is activated by ABA treatment and several crops transformed with the *HVA1* gene showed improved drought and salt tolerance (Nguyen and Sticklen, 2013). *HVA1* gene was transformed to spring wheat by gene gun and its over-expression under maize ubiq1 promoter resulted in improved biomass production, WUE, drought, and salt tolerance due to activation of ABA signaling (Sivamani et al., 2000). Field evaluation of transgenic wheat with *HVA1* for six cropping seasons at four locations (USA and Egypt, both irrigated and rainfed area) showed stable yield, higher WUE and relative water content (RWC). These tolerance traits were directly correlated with the expression of *HVA1* gene in transgenic plants (Bahieldin et al., 2005). It is important to note that to date, the research station has not released it as a variety (Hayes and Xue, 2014).

#### Drought Signaling by Introducing Other Genes

Transformation of wheat with *SNAC1* gene under the control of ubiquitin promoter showed enhanced salinity and drought tolerance at seedling stage in lab conditions. Transgenic plants showed higher biomass, RWC, and chlorophyll content. Expression studies of *SNAC1* by qPCR showed over-expression of sucrose phosphate synthase, type 2C protein phosphatases and 1-phosphatidylinositol-3-phosphate-5-kinase genes, which are involved in ABA signaling (Saad et al., 2013). Transgenic wheat with cDNA of alfalfa aldose reductase recipient gene, involved in antioxidant defense and exhibited 1.5–4.3 times more detoxification of aldehyde substrate and 12, 26, and 41% increase in green biomass production in three separate transgenic lines, resulting in enhanced drought tolerance (Fehér-Juhász et al., 2014). Although the transfer of *DREBs, HVA1*, and *NAC* have resulted in enhanced drought tolerance by improving signaling pathways, there are no studies to date that show whole downstream signaling cascade improved in transgenic plants.

## Genomics Based Improvement of Drought Signaling

Although a reference whole genome sequence has not been reported for wheat to date, efforts have been put to identify potential genomic regions carrying genes for drought signaling pathways. QTLs, miRNAs, and genome editing systems (e.g., CRISPR/Cas system) are major genomics based methods applied to discover and manipulate related genomic regions. The role of these approaches in characterizing genes involved in drought signaling in wheat is discussed below.

### QTL Mapping for Drought Signaling Genes in Wheat

Drought tolerance is a complex and quantitative trait encoded by many genes, and thus, the identification of genomic regions carrying genes for drought signaling is important. Doubled haploids (DHs), F_2_ populations (Ibrahim et al., 2012), recombinant inbred lines (RILS), near isogenic lines (NILS) are suitable populations for QTL mapping (Budak et al., 2013b). In wheat, the QTLs for yield and yield related traits in drought, and biomolecules involved in signaling pathways have been mapped. Inheritance of ABA accumulation and distribution in plants is not simple and several genes/QTLs are involved in it. A major QTL for ABA production was mapped on the long arm of the 5A chromosome between *Xpsr575* and *Xpsr426* loci (8 cM from *Xpsr426*) in single chromosome substitution line and their subsequent F_2_ and DHs populations. Substitution lines were developed by from hybridization of low and high ABA producing Chinese Spring’ and ‘SQ1’ genotypes (Quarrie et al., 1994). This QTL showed strong linkage with *Dhn1*/*Dhn2* locus depicting a direct association between ABA accumulation and early flowering based drought tolerance in wheat (Ibrahim et al., 2012). Nine QTLs were mapped in wheat in response to exogenously applied ABA, SA, JA, and ethylene, suggesting the presence of potential genes involved in signaling in these regions (Castro et al., 2008).

Several QTLs linked to ABA accumulation in leaves were mapped in an F_2_ population developed from the cross of contrasting genotypes for ABA production. But one novel QTL was linked to both higher ABA content and smaller leaf size due to genetic linkage between the genome regions. The QTL location was a homoeolog of the major wheat gene *Vrn1* that code for number of tillers, ABA accumulation and leaf size (Quarrie et al., 1997). A major QTL for ABA production was mapped on chromosome 6D in an F_2_ population developed from contrasting SHs. This QTL region has several genes for ABA responsiveness, seed dormancy, and regulation of LEA proteins that protect the cell machinery under dehydration stress (Iehisa et al., 2014). A major QTL for higher grain yield (21%), chlorophyll content and wider flag leaf on 7A chromosome was mapped in a DH wheat line by using psp3094 SSR. Exogenous ABA application activated this QTL suggesting that genes in this region might be involved in ABA signaling (Quarrie et al., 2007). Four homologs of *Arabidopsis* ABA signaling genes (*TmABF, TmVP1, TmERA1*, and *TmABI8*) were mapped in a wheat RILs population derived by crossing *T. boeoticum* and *T. monococcum*. The location of these QTLs was chromosome 3Am, 4Am, and 5Am (Nakamura et al., 2007).

Seven QTLs for ABA production in response to drought were identified on chromosomes 2A, 3A, 1B, 7B, and 5D in an F_4_ population at 33% field capacity. The most important QTLs for ABA content were mapped on chromosomes 3B, 4A, and 5A on the marker location of *Wmc96, Trap9*, and *Barc164* (Barakat et al., 2015). In another study, five major QTLs for ABA responsiveness were identified on chromosomes 1B, 2A, 3A, 6D, and 7B in a wheat RILs population. A QTL located on chromosome 6D contributed 11.12% to variation for ABA against 5–8% contribution by other QTLs. Expression analysis showed allelic differences in QTLs for three ABA responsive Cor/LEA protein coding genes, *Wrab15, Wdhn13*, and *Wrab17*. The expression of these genes was influenced by QTLs present on chromosomes 2A, 7B, and 6D in ABA treated seedlings (Kobayashi et al., 2010). In conclusion, several QTLs for ABA production and downstream signaling pathways have been mapped in wheat (**Table 3**) but most of studies have not focused further on this aspect. We recommend the use of functional genomics tools along with QTLs to identify the genes located in QTL regions.

**Table 3:** Quantitative trait loci (QTLs) mapped for drought signaling molecules in wheat.

Chromosome location	Function	Reference
MAPMAKER QTL; 5A	Located between Xpsr575-Xpsr426, ABA production	Quarrie et al., 1994
2A	ABA production, smaller leaf size, homoeolog of wheat gene *Vrn1* that controls number of tillers and ABA accumulation	Quarrie et al., 1997
7A	Activated on exogenous ABA application, involved in ABA signaling	Quarrie et al., 2007
3Am, 4Am, 5Am	QTLs for ABA signaling genes (*TmABF, TmVP1, TmERA1*, and *TmABI8)*	Nakamura et al., 2007
6A	Located at *Xgwm459* and *gwm334a region*, activated by exogenous ABA, salicylic acid (SA), jasmonic acid (JA), and ethylene application, contains signaling genes	Castro et al., 2008
1B, 2A, 3A, 6D, 7B	ABA responsiveness, regulate expression of ABA responsive LEA protein coding genes, i.e., *Wrab15, Wdhn13*, and *Wrab17*	Kobayashi et al., 2010
MAPMAKER QTL; 5A	Linked to *Dhn1/Dhn2* genes, early flowering based drought tolerance	Ibrahim et al., 2012
6D	ABA production and signaling, seed dormancy, regulation of LEA proteins	Iehisa et al., 2014
3A, 1B, 4A, 5A, 5D, 7B	Enhanced ABA production, located at marker locations of *Wmc96, Trap9*, and *Barc164*	Barakat et al., 2015

### miRNAs Involved in Drought Signaling and Tolerance

Extensive application of NGS platforms has greatly contributed in identification of 20–22 nt short non-coding RNAs called as miRNAs that play regulatory roles in many processes (Budak et al., 2015a). miRNAs bind to their target transcripts through complementary base pairing, and either direct the cleavage of the target or repress its translation, leading to the decreased expression of the target transcript. Thus, miRNAs can act both at the transcriptional or post-transcriptional levels. miRNA mediated gene-silencing mechanism regulates the expression of plant hormones, TFs, and other developmental/stress signaling pathways (Curaba et al., 2014). Gene silencing involved in plant stress regulation is also mediated by naturally occurring small RNAs (siRNAs; Lu et al., 2012), and complementary double-stranded RNA (dsRNA) generated from inverted repeat (IR) transgenes (Frizzi and Huang, 2010). Post transcriptional gene silencing is carried out by miRNAs and virus-derived small interfering RNAs (vsiRNAs) which helps in discovering gene functions and developing crops with improved stress tolerance (Feng et al., 2013). miRNAs are important regulators in plant drought signaling because their target genes have key roles in metabolism and signal transduction (Yin et al., 2014).

The miRNA gene transcripts ‘*MIR*’ are spatially and temporally influenced by cellular signaling factors, particularly plant hormones such as ABA under stresses (Jung et al., 2015). Some conserved plant miRNAs such as miR159 (*Triticum*, French bean, cotton, maize), miR164 (*Triticum, Brachypodium*, sugarcane), miR172 (*Triticum, Arabidopsis, Brachypodium, Oryza*, cotton) and miR393 (*Triticum, Oryza, Medicago, Pinguicula, Arabidopsis*) control the expression of key TFs which regulate development and signaling pathways (Gupta et al., 2014). miRNAs are involved in various drought related cellular pathways, including auxin signaling, ABA response, antioxidant defense, osmoprotection, cell growth, respiration, and photosynthesis, e.g., miR169 shows bread wheat specific differential expression under drought (Ding et al., 2013). Several signaling genes (ARF, *MYB33, MYB101, TIR1, AGO1*,) and growth regulation factors (GRF) are targeted and regulated by drought responsive miRNAs and *DREBs* (Covarrubias and Reyes, 2010). Most of the miRNAs have their specific putative targets which lead to regulation of specific genes/TF involved in signaling/tolerance mechanisms. In such a study, various bread wheat based miRNAs and their targets (shown in parentheses) were identified as tae-miR159a,b (MYB3), tae-miR159c-5p (Dihydro-flavonoid reductase-like protein), tae-miR171f (sensor histidine kinase), tae-miR395i (ATP sulfurylase), tae-miR156k (SBP), tae-miR166l-5p (FAM10 family protein), tae-miR168b (dehydrogenase/reductase), tae-miR444c.1 (MADS-box TF), tae-miR1432 (mitochondrial phosphate transporter), tae-miR160a (ARF), tae-miR164b (NAC), tae-miR166h (HD-ZIP4), tae-miR169d (CCAAT-box TF), tae-miR319c (Acyl-CoA synthetase), tae-miR393b,i (TIR1), tae-miR396a,c,g (GRF), tae-miR444d (IF3), tae-miR827-5p (finger-like protein). The above mentioned miRNAs regulated the expression of their targeted TFs/genes thus playing key roles in drought tolerance mechanism (Ma et al., 2015).

Similarly, increased expression of miR156 in *T. dicoccoides* targets the SBP TFs and promoted flowering while miR398 targets copper superoxide dismutases, cytochrome C oxidase, and regulates ROS production under drought stress. Increased expression level of miR1432 targets calcium-binding EF which activates signal transduction pathways. Other important drought responsive miRNAs in *T. aestivum* and *T. dicoccoides* are miR396, miR528, miR6248 (Kantar et al., 2011b; Budak et al., 2015b), miR1435, miR5024, and miR7714 (Akpinar et al., 2015). On the other hand, miR166 exhibits decreased expression in *T. dicoccoides* under drought which targets HD-ZIP3 TF and plays role in developmental while miR171 targets GRAS TF and is significant in abiotic stress responses (Kantar et al., 2011b). A summary of miRNAs involved in drought response and signaling is given in **Table 4**. For a further comprehensive reading on drought responsive miRNAs, we would recommend our reader to go through other articles from our group (Lucas et al., 2011b; Budak et al., 2015a,b). From above discussion, it is evident that miRNAs have a key role in regulating drought tolerance pathways and should be exploited for wheat improvement.

**Table 4:** Wheat miRNAs involved in drought stress signaling.

miRNAs	Differential expression under drought	Target genes related to signaling	Function	Reference
miR159a-5p	↑	MYB TF, GAMYB1 GAMYB2, genes for oligopeptide transport	Auxin Signaling, Oligopeptide transporter	Liu et al., 2013; Gupta et al., 2014
miR159a,b	↓	WRKY TF; MYB3; alkaline phosphatase, cytochrome P450, Mob1-like and TLD protein	Signaling	Ma et al., 2015
miR160a	↑	HSP 70; ARF; tetratrico peptide repeat (TPR)	Stress adaptation	Liu et al., 2015
miR164	↑	NAC domain TF, phytosulfokines, sHSPs 17; NAC TF; genes involved in MAPK signaling pathways	Signaling pathway, oxidative stress response	Gupta et al., 2014
miR166h	↑	Class III HD-ZIP protein 4	Stress response, phytohormones	Ma et al., 2015
miR167	↑	Dnaj heat shock n-terminal domain-containing protein	Auxin signaling pathway, developmental response	Liu et al., 2015
miR168	↑	Argonaute	Signal transduction, stress response	Gupta et al., 2014
miR169d	↓	CCAAT-box TF	ABA-responsive transcription, drought tolerance	Ma et al., 2015
miR171f	↓	Sensor histidine kinase	Stress response	Li et al., 2013
miR172	↑	Apetala2-like TF, ARF, helix-loop-helix DNA-binding protein	Signaling Pathway, stress response, development	Gupta et al., 2014
miR172a,b	↓	Floral homeotic protein, AP2 TFs	Flower development signaling pathway	
miR319	↓	MYB3, Acyl-CoA synthetase	Abiotic stress tolerance	Zhou et al., 2010
miR393	↑	bHLH TF, transport inhibitor response 1/auxin F-box	Signaling pathway, genes in auxin signaling, basal defense	Gupta et al., 2014
miR395i	↓	ATP sulfur lyasas and sulfur transporters	Abiotic stress	Sunkar et al., 2005
miR397	↑	Ice1 (inducer of CBF expression 1) TF, laccase	Response to water deprivation	Gupta et al., 2014
miR398	↑	COX, Superoxide dismutase (SOD) gene family	Respiration pathway	Kantar et al., 2011b
miR474	↓	PPR, protein kinase, kinesin, Leucine-rich repeat	Unknown	Kantar et al., 2011b
miR1029	↑	Apetala2-like TF, DREB TF	Signaling pathway abiotic stress	Gupta et al., 2014
miR1432	↑	Mitochondrial phoshphate transporter	–	Ma et al., 2015

*↑, increased expression levels; ↓, decreased expression levels.*

### CRISPR/Cas Genome Editing System

In addition to ZEN and TALEN, an efficient bacterium based genome editing system called the Clustered Regulatory Interspaced Short Palindromic Repeats (CRISPR), with associated protein Cas (CRISPR/Cas system) has emerged. The CRISPR are loci with variable short spacers interspersed by short repeats, later transcribed into non-coding RNAs (ncRNA). This ncRNA then forms a complex with the Cas and guides the complex to slice complementary target DNA. The development of single guide RNAs (sgRNAs) which are fusions of essential parts of *trans*-activating crRNA (tracrRNA) and the sgRNA of CRISPR RNA (crRNAs) proved to be an essential improvement in adopting the CRISPR-Cas system for targeted editing of complex eukaryotic genomes (Jiang et al., 2014). Following *Arabidopsis*, the system has also been demonstrated in rice and other crop plants. In protoplasts of bread wheat cultivar Kenong199, an ortholog of the barley MLO protein, *TaMLO* gene was targeted and showed high INDEL frequencies of 26.5–38.0%. The number of unique sgRNA target candidates generated on average were 21 per cDNA of either A or D genomes. The mean mutagenesis frequency in protoplasts was 28.5% with the transformation efficiency of 70–80% (Shan et al., 2013). The ability of this system to delete large DNA segments stably is valuable in wheat genomics given its large genome size and complexity. Development of transgenic wheat cultivars with stable drought tolerance through targeted genome editing will potentially revolutionize crop breeding. To date, the use of this system in engineering abiotic/drought stress tolerance or signaling has not been reported; however, in the future, it may prove to be a valuable tool in discovering the functions of signaling pathway components.

## Functional Genomics for Discovering Drought Signaling Molecules

**FIGURE 1:**
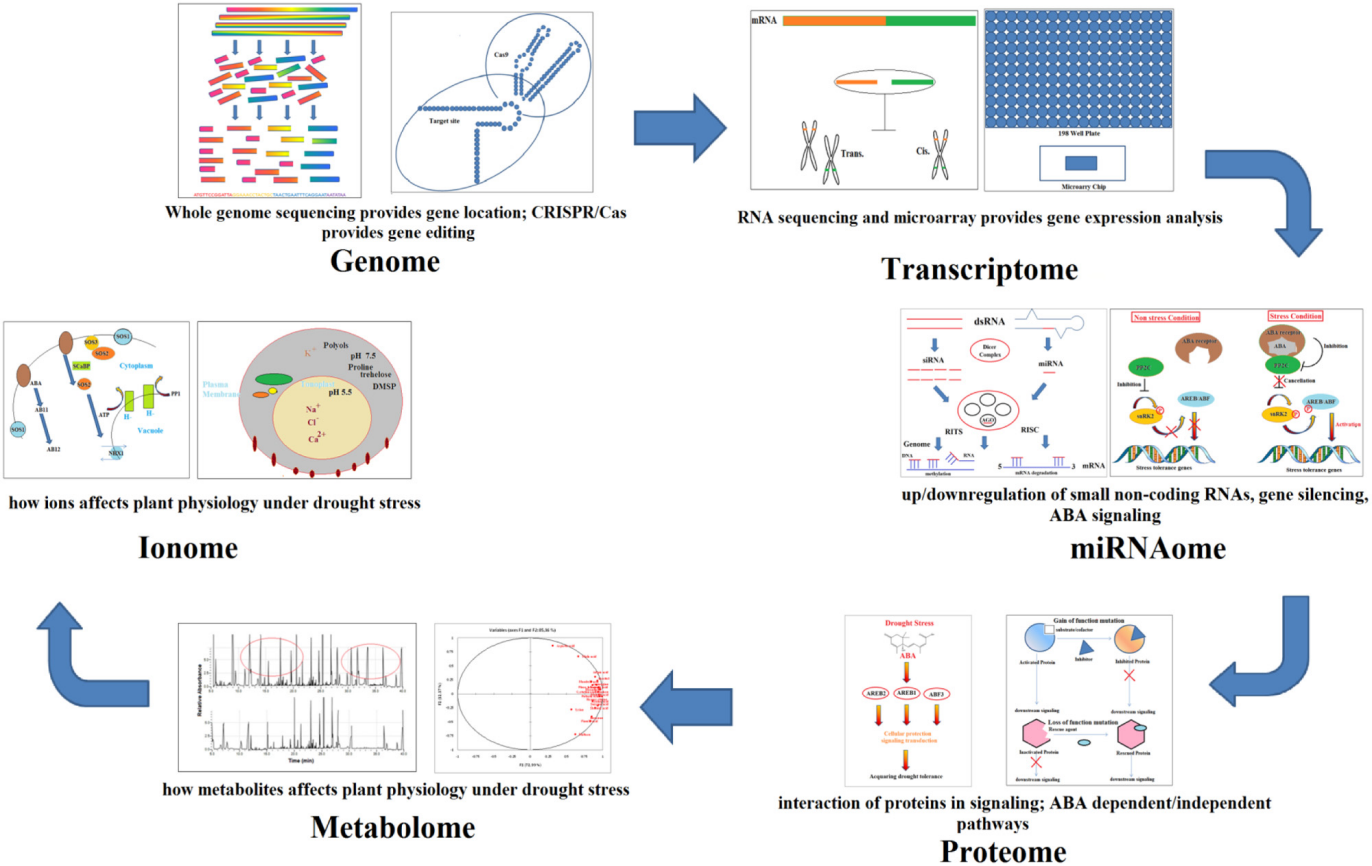
**Schematic diagram showing flow of information between functional genomics techniques for studying signaling pathways in plants**.

In recent years, the use of functional genomics tools has considerably increased in elucidating abiotic stress tolerance in plants. These methods include transcriptomics, metabolomics, proteomics, and ionomics and are capable of discovering and characterizing the expression of genes or other molecules in drought with high accuracy and efficiency. These highly sensitive tools can perform the spatial analysis of tissues which helps to understand the drought tolerance mechanisms, see **Figure 1** (Ergen et al., 2009). We have summarized the advances in OMICS for discovery of drought signaling molecules below

### Transcriptomics for Identification of Drought Signaling Pathways

Transcriptomics is the study of whole set of RNA transcripts (transcriptome) produced by an organism under certain conditions, like drought. Microarrays were used first for profiling transcripts in response to various stresses, but polyploidy has limited the number of studies in wheat. Microarray based analysis became more common after the commercial release of wheat Affymetrix Gene Chip^®^ (Santa Clara, CA, USA) which contained over 55,000 probes for wheat transcripts (Nair et al., 2012). Ergen et al. (2009) identified several novel genes and TFs involved in ABA, ethylene, and IP3 dependent signaling by Affymetrix Gene Chip^®^ based spatial profiling of root and leaf tissues in wild emmer wheat. In leaf tissue, glutamine-dependent asparagine synthetase, a putative ATP-binding protein, homeo-domain TF Hox22 homolog (linked to LEA3 proteins), carotenoid producing 9-*cis*-epoxycarotenoid dioxygenase, proline-rich protein precursor and many proteinase inhibitors of Bowman Birk type showed highest increased expression levels under drought. While, germin-like protein, protein degrading cysteine proteinase precursors, bZIP TFs, cytochrome P450, OsRR9 homolog (a signal receiver domain), *LOL1* protein, and G–C content rich motif binding MYB like TF RADIALIS exhibited decreased expression levels. In root tissues, several dehydrins, LEA/COR protein *WRAB1*, putative lipases, a Rab GTPase homolog, several cold regulated proteins, 12-oxophytodienoic acid reductase, a cold shock protein A-2, MYB-domain Hv1 TF, glutathione transferase (ROS scavenger), PRP homologs and *WCOR719* (actin depolymerization factor) showed the highest increased expression levels. In contrast, many HSPs, RmlC-type cupin domain, B12D proteins, two nodulin 93 proteins, RING-H2 finger protein and GTP-binding EF exhibited decreased expression levels (Ergen et al., 2009).

Microarray gene chip based transcriptomic analysis of *TAM111* and *TAM112* wheat genotypes identified 123 genes for production of ABA, JA, auxin, cytokinin, brassinosteroid, gibberellins, and ethylene, and signaling pathways involving these hormones. These transcripts showed differential expression at grain filling stage and transcripts for ABA biosynthesis exhibited increased expression levels in both genotypes. Two transcripts similar to *PDR12* (coding for ABA transporter), transcripts for auxin, LEAs, dehydrins, HSPs, aquaporins, and redox homeostasis showed decreased expression levels. Transcript analysis revealed a key role of ABA in regulation of transcripts and physiological changes linked with drought adaptation. Higher leaf ABA production showed strong association with higher yield and biomass under field drought which reduced the stomatal conductance and was linked to elevated transcript changes in flag leaf (Reddy et al., 2014). Low chip-to-chip variation, high reproducibility and probe density of Affymetrix Gene Chip^®^ are its advantages but a major disadvantage of high relative cost has limited large scale studies. Therefore, there is dire need for more reliable and cost effective methods to study the complex drought signaling pathways.

Deep sequencing (RNA-Seq) based transcriptomics has made it a cost effective and powerful tool. There were unique transcripts found in *T. dicoccoides*, and *T. durum* under drought that they were engaged in drought signaling pathways (Akpinar et al., 2015). It can be concluded that RNA-Seq based transcriptomic analyses identified various drought signaling genes such as bZIP, TdNAC, and MYB genes which can be exploited in future studies.

### Proteomics for Identification of Drought Signaling Pathways

Though transcriptomics provides the gene expression profiles, these profiles do not necessarily reflect protein levels as some transcripts may not be translated. Proteomics analysis provides clues into the actual fluctuations of the protein levels involved in signaling, regulatory, and enzymatic functions encoded by genome through transcripts. Advanced bioinformatics tools have helped to identify, characterize, and annotate novel proteins (Colmsee et al., 2012). The proteomic analysis of two wheat genotypes having contrasting drought tolerances showed that among differentially expressed proteins, 26% were involved in carbohydrate metabolism, 23% in detoxification and defense, and 17% in storage proteins. In drought, WD40 repeat protein, catalase isozyme 1, LEAs, Triticin precursor, sucrose synthase, and alpha amylase inhibitors exhibited increased expression levels in tolerant and decreased expression level in sensitive cultivar. On the other hand, ascorbate peroxidase, small and large subunit ADP glucose pyrophosphorylase and G-beta like protein showed decreased expression levels in sensitive cultivar (Jiang et al., 2012). Our group performed proteomics analysis using two wild emmer wheat varieties (TR39477 and TTD22), and one durum wheat cv. (Kızıltan). After 9 days of drought exposure, 75 differentially expressed proteins were detected with many being common to all three wheat genotypes, e.g., manganese superoxide dismutase (MnSOD), a glutathione transferase showed increased expression in the durum wheat (Budak et al., 2013a).

Alvarez et al. (2014) identified 1656 proteins and two unique peptides in wheat through proteomics analysis by using roots of from drought tolerant (Nesser) and sensitive (Opata) varieties for ABA-responsiveness. Important signaling proteins including monomeric G-proteins and their regulators, two lipoxygenases, K channel β subunits, a plasma membrane proton ATPase, calnexin, and an elicitor-induced protein showed an increased response to ABA in drought tolerant cultivar and *vice versa* in the sensitive. Though signaling protein 14-3-3 homologs exhibited increased expression in the drought tolerant cultivar, they remained unchanged in the sensitive one. Out of 151 ABA-responsive proteins, 100 showed increased expression levels but the rest showed decreased expression levels. An interesting finding was the abundance of multiple porin proteins and β-expansin precursor in the sensitive cultivar suggesting that the cell wall structure and membrane permeability might have influenced different adaptation to drought in both cultivars. Furthermore, six LEA proteins and several phosphatases were also identified in both cultivars (Alvarez et al., 2014).

### Metabolomics for Identification of Drought Signaling Pathways

Metabolites are considered as signaling molecules as they are associated with physiological processes and are exported from each organelle to cytoplasm in the form of retrograde signals. Gas chromatography mass-spectrometry (GC–MS), liquid chromatography mass-spectrometry (LC–MS), capillary electrophoresis mass-spectrometry (CE–MS), and nuclear magnetic resonance (NMR) are the major analytical tools in metabolomics to detect, identify, and analyze small molecules. Initially, the analysis of metabolites was limited to a few compounds having major roles in drought tolerance. But, advances in these methods have enabled us to identify a wider range of metabolites produced under a specific condition. Metabolite profiling is a powerful tool to characterize genotype or phenotype of an organism for dissecting novel signaling pathways (Xiao et al., 2012). Plants accumulate compatible solutes to protect them from drought and oxidative stress for survival. GC–MS based metabolite profiling in moss *Physcomitrella patens* showed accumulation of compatible solutes in response to drought stress (Zhan et al., 2014). In wheat, levels of proline, tryptophan, and the branched chain amino acids leucine, isoleucine, and valine increased under drought in tolerant cultivars but organic acid levels decreased (Krugman et al., 2011). There are various examples where metabolites act as intracellular signals, e.g., in response to cytosolic sugar levels, trehalose 6-phosphate (T6P) enhances the redox transfer to AGPase, mediated by thioredoxin, mainly depending on metabolite balance between the chloroplast and cytosol. Metabolite profiling for elucidating signals in plants has been characterization in form of methyl-erythritol-cyclo-diphosphate (MECD; Xiao et al., 2012).

### Ionomics for Identification of Drought Signaling Pathways

Ionomics is a high throughput analysis of the ion composition in an organism under a certain condition. It has immense applications in forward and reverse genetics, screening of mutants, finding mechanisms of ion uptake, compartmentalization, transport, and exclusion, thus helps to understand the mechanisms of drought and other abiotic stresses in plants (Shelden and Roessner, 2013). In wheat, ionomics studies under drought stress have not been reported yet. Together, ionomics and other genomics data can provide a complete picture of cellular changes against drought, enabling a thorough understanding of the underlying mechanisms of tolerance (Colmsee et al., 2012). Furthermore, ionomics can help to identify novel genes coding for ions by utilizing phenotypic and genotypic data obtained from mapping populations. It can thus help in understanding the gene networks controlling the ion accumulation at different growth stages under drought stress (Satismruti et al., 2013). It should be noted that ionomics is a relatively new functional genomics tool with limited number of studies available, but spatial and highly sophisticated ion profiling will be a key in the future to understand the signaling pathways for drought tolerance.

## Systems Biology Approaches to Discover Signaling Pathways

System biology is a recent, fast growing and comprehensive analytical approach in life sciences to discover the control and regulation of intracellular biological systems, biochemical cycles and pathways in plants under different environmental stresses. Several computational studies have been involved, from few decades, to find the biochemical function of metabolites and small biomolecules responsible for biochemical pathways under drought stress (Gutiérrez et al., 2005). Most of the experimental methods are being used as the part of system biology including various target and untargeted metabolite analysis approaches used to identify the drought specific metabolites in different species of plants. GC–MS is one of the several approaches, was used to find metabolic compounds differentially expressed in tolerant (Excalibur and RAC875) and sensitive (Kukri) bread wheat cultivars under drought stress environment (Bowne et al., 2012). Another finding was aspartate-derived synthesis of few amino acids including lysine, methionine, and threonine in *Arabidopsis thaliana* done also explains the advantageous use of system biology approach, model based on measured kinetic parameters (Curien et al., 2009). Thus, system biology helps to understand the relationship and inter-connection among various types of bio-molecules involved in a certain tolerance mechanism.

## Conclusion

**FIGURE 2:**
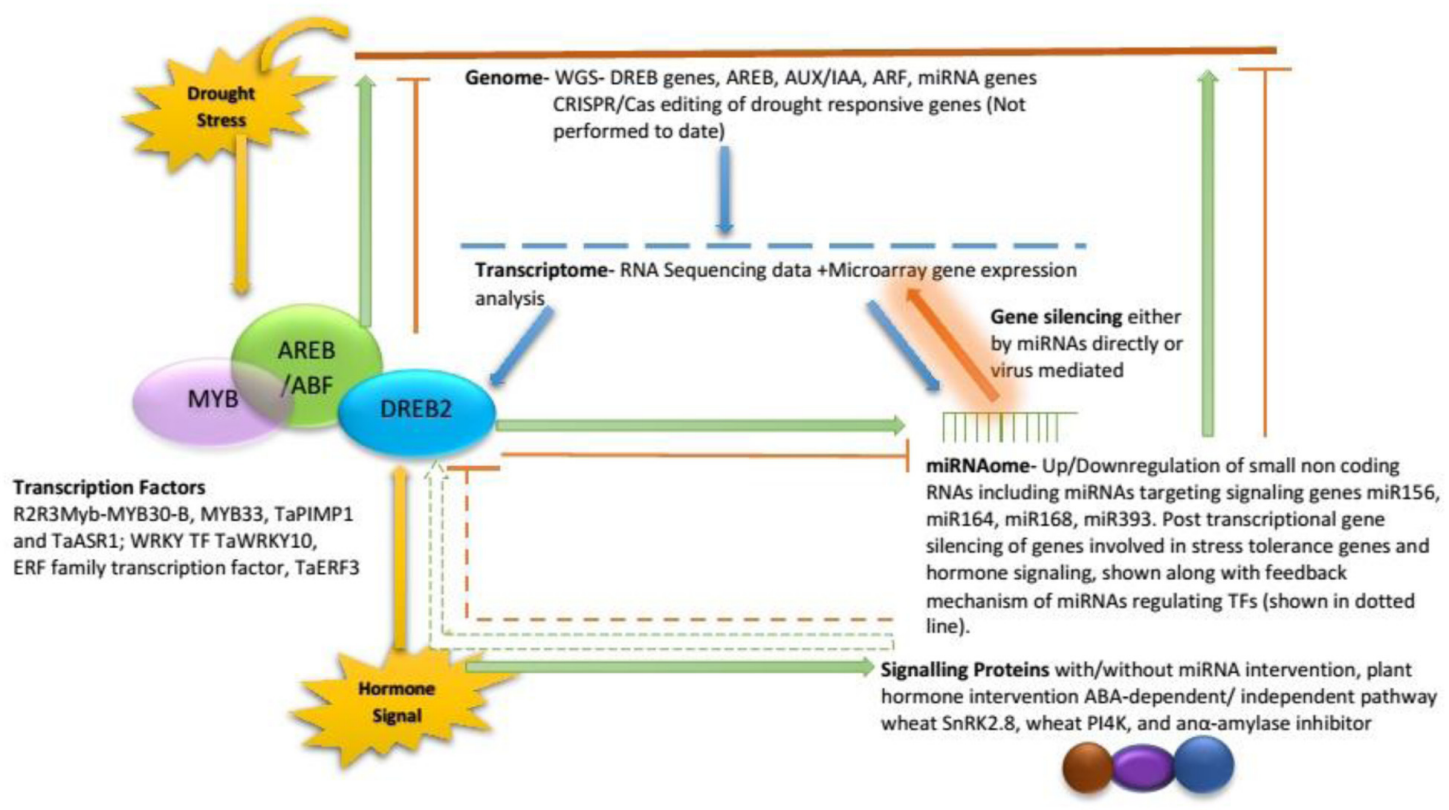
**Putative stress signaling pathway in wheat where functional genomics contribute**.

Significant wheat yield losses due to drought lower farmers’ income, food availability and ultimately affect the economy of various countries. Few drought tolerant wheat varieties have been developed as most of breeders have selected plants on the basis of morphological traits and ignored physiological basis of drought tolerance. Lost genetic variation for drought tolerance has been patched up by using wild wheat *T. dicoccoides* and *A. tauschii* in crossing and synthetic wheat showed improved drought tolerance. The improvement has been measured in terms of novel genes, QTLs, ESTs, and SNPs, e.g., three LEA protein coding genes (*Wrab18, Wrab17, Wdhn13*) involved in ABA signaling pathway were identified in SHs. Proteomics analysis identified ABA 8′-hydroxylase, MPK6, dehydrin, 30S ribosomal protein S1, and a 70 kDa HSP involved in ABA signaling in wild emmer wheat. In recent years, MAS have been used to select the plants which make the cultivar development process less time consuming. SNP markers have identified *DREB-B1, DREB1A, ERA1-B, ERA1-D, 1-FEH-A, 1-FEH-B, WRKY1, TaSnRK2.8*, and *HKT-1* genes in wheat. Many QTLs for ABA production, SA, JA, ethylene, and ABA based signaling, and QTL having genes for regulating other signaling genes (*TmABF, TmVP1, TmERA1* and *TmABI8, Wrab15, Wdhn13*, and *Wrab17)* have also been mapped. Transgenic approach provides the benefit of speedy gene transfer without any genetic barriers. Transformation of wheat with *GmDREB, GhDREB, DREB1A, HVA1, SNAC1*, and aldose reductase genes improved drought signaling and tolerance. Similarly, various miRNAs showed differential expression in drought and enhanced or silenced the expression of genes involved in drought signaling. However, the signaling genes, miRNAs, TF, etc., don’t not express in isolation but interact with each other in signaling pathways (**Figure 2**).

In recent years, functional genomics has emerged as a power tool to identify the molecules involved in drought signaling pathways. Transcriptomics analyses identified various genes including Hox22, bZIP TF, dehydrins, *WRAB1, WCOR719, HSPs*, LEA, *TaWRKY17, TaWRKY16, TaWRKY24, TaWRKY19-C, TaWRKY59, TaWRKY82, TaWRKY61, TaWLIP19, TaWRKY10, TaNAC69*, and *TaMYB33* for drought signaling pathways. Proteomics and metabolomics have identified several proteins (Monomeric G-proteins, lipoxygenases, potassium channel β subunits, calnexin, LEAs, phosphatases) and metabolites (Proline, tryptophan, leucine, isoleucine, valine) involved in drought signaling as summarized in **Table 5**. In this way, highly efficient functional genomics tools have helped in identifying several important genes which can be exploited by breeders to develop drought tolerant wheat cultivars in futures. Similarly, genome-editing system CRSPR/Cas will be valuable in future for better understanding of drought tolerance mechanisms due to its ability to modify the genome. Study of miRNAs in future is also important in future as they are key regulators of signaling pathways.

**Table 5:** Functional genomics studies for identification of drought signaling molecule in recent years.

Functional genomics tool	Drought signaling genes/molecules/mechanism identified	Reference
Transcriptomics	Hox22 TF linked to LEA3 proteins, ABA inducible LEA *WRAB1*, Dehydrins, Proline-rich protein precursor, Asparagine synthetase, 9-*cis*-epoxycarotenoid dioxygenase, Rab GTPase homolog, A-2 cold shock protein, MYB-domain Hv1 TF, glutathione transferase, *WCOR719*	Ergen et al., 2009
Transcriptomics	Genes for ABA, JA, auxin, cytokinin, brassinosteroid, gibberellins, and ethylene production and drought signaling based on these hormones. Transcripts for *PDR12*, auxin, LEAs, dehydrins, HSPs, aquaporins, redox homeostasis, and reduced stomatal conductance	Reddy et al., 2014
Proteomics	WD40 protein, catalase isozyme 1, LEA and alpha amylase inhibitors, ascorbate peroxidase, G-beta like protein, triticin precursor, sucrose synthase	Jiang et al., 2012
Proteomics	Ribulose-1,5-bisphosphate carboxylase large subunit, OsI_16800 protein, SORBIDRAFT_09g029170 protein, polyamine oxidase, Os02g0101500, Os03g0786100, Ferredoxin-NADP(H) oxidoreductase, Os03g0786100, Glutathione transferase, Mn superoxide dismutase, Cold regulated proteins	Budak et al., 2013a
Proteomics	Monomeric G-proteins and their regulators, lipoxygenases, K channel β subunits, plasma membrane proton ATPase, calnexin, an elicitor-induced protein, porin proteins, β-expansin precursor, LEAs, phosphatases	Alvarez et al., 2014
Proteomics	8′-hydroxylase, MPK6, dehydrin, 30S ribosomal protein S1, retrotransposon protein, 70 kDa HSP, thioredoxin peroxidase, ascorbate peroxidase, Cu/Zn superoxide dismutase	Yang et al., 2015
Metabolomics	Proline, tryptophan, leucine, isoleucine, and valine, organic acids	Krugman et al., 2011
Metabolomics	Trehalose 6-phosphate (T6P) promotes thioredoxin-mediated redox transfer to AGPase and MECD helped in elucidating signaling in plants	Xiao et al., 2012

## Conflict of Interest Statement

The authors declare that the research was conducted in the absence of any commercial or financial relationships that could be construed as a potential conflict of interest.
